# Association between hemoglobin level and mortality in patients undergoing maintenance hemodialysis: a nationwide dialysis registry in Japan

**DOI:** 10.1007/s10157-025-02632-9

**Published:** 2025-02-11

**Authors:** Takaaki Kosugi, Takeshi Hasegawa, Takahiro Imaizumi, Hiroki Nishiwaki, Hirokazu Honda, Yasuhiko Ito, Kazuhiko Tsuruya, Masanori Abe, Norio Hanafusa, Takahiro Kuragano

**Affiliations:** 1 Department of Nephrology, Nara Medical University, 840 Shijo-Cho, Kashihara, Nara 634-8521 Japan; 2 Institute of Clinical Epidemiology (iCE), Showa University, Tokyo, Japan; 3 Department of Hygiene, Public Health, and Preventive Medicine, Graduate School of Medicine, Showa University, Tokyo, Japan; 4 Department of Nephrology, Graduate School of Medicine, Showa University, Tokyo, Japan; 5 Showa University Research Administration Center, Showa University, Tokyo, Japan; 6 Center for Innovative Research for Communities and Clinical Excellence, Fukushima Medical University, Fukushima, Japan; 7 Department of Healthcare Epidemiology, School of Public Health in the Graduate School of Medicine, Kyoto University, Kyoto, Japan; 8 Department of Advanced Medicine, Nagoya University Hospital, Nagoya, Japan; 9 Department of Nephrology, Nagoya University Graduate School of Medicine, Nagoya, Japan; 10 Division of Nephrology, Department of Medicine, University of Illinois at Chicago, Illinois, USA; 11 Division of Nephrology, Department of Medicine, Showa University Fujigaoka Hospital, Yokohama, Japan; 12 Department of Nephrology and Rheumatology, Aichi Medical University, Nagakute, Japan; 13 Division of Nephrology, Hypertension and Endocrinology, Department of Internal Medicine, Nihon University School of Medicine, Tokyo, Japan; 14 Department of Blood Purification, Tokyo Women’s Medical University, Tokyo, Japan; 15 Division of Kidney and Dialysis, Department of Internal Medicine, Hyogo College of Medicine, Nishinomiya, Japan

**Keywords:** Anemia, Hemodialysis, Hemoglobin, JRDR, Mortality

## Abstract

**Background:**

The optimal hemoglobin (Hb) range in patients undergoing hemodialysis remains controversial. We aimed to investigate the association between Hb levels and mortality in patients undergoing hemodialysis and explore the potential factors modifying this association.

**Methods:**

This observational study utilized a nationwide database from the Japanese Renal Data Registry spanning from 2019 to 2021. This study included 265,779 patients undergoing hemodialysis thrice a week. The association between Hb levels and all-cause and cause-specific mortality was investigated using Cox regression analysis. The nonlinear relationship between Hb levels and outcomes was investigated using restricted cubic spline analysis.

**Results:**

During a median follow-up period of 24 months, 45,734 patients died. Compared to the reference Hb category of 10–10.9 g/dL, the risk of all-cause mortality was higher in the Hb categories of < 9.0, 9.0–9.9, and ≥ 13 g/dL with adjusted hazard ratios (95% confidence intervals) of 1.24 (1.20–1.29), 1.09 (1.06–1.12), and 1.19 (1.14–1.25), respectively. Restricted cubic spline analysis also showed a U-shaped relationship between Hb level and mortality. The subgroup analysis indicated that the Hb category of 12.0–12.9 g/dL was associated with increased mortality risk in patients with a dialysis vintage of ≥ 10 years and those with a history of cerebral infarction.

**Conclusion:**

Hb levels of < 10.0 and ≥ 13.0 g/dL were significantly associated with an increased mortality risk compared to an Hb level of 10.0–10.9 g/dL in patients undergoing hemodialysis.

**Supplementary Information:**

The online version contains supplementary material available at 10.1007/s10157-025-02632-9.

## Introduction

Anemia is a common complication in patients undergoing maintenance hemodialysis (HD) and is associated with adverse outcomes, including cardiovascular disease (CVD) and mortality [1–3]. The management of anemia in patients with chronic kidney disease (CKD), including those undergoing HD, has dramatically improved since the introduction of erythropoiesis-stimulating agents (ESAs) in clinical practice. However, several trials showed that targeting normal or high levels of hemoglobin (Hb) through ESA therapy did not improve the primary outcomes of HD patients [4, 5]. To date, several guidelines for anemia in patients with CKD have focused on defining the optimal or target Hb levels [6]. The Kidney Disease Improving Global Outcomes (KDIGO) guidelines for anemia published in 2012 recommended the administration of ESAs to maintain an Hb level of > 9 g/dL without exceeding 11.5 g/dL [7]. Meanwhile, the Japanese Society for Dialysis Therapy (JSDT) revised its guideline in 2015 and set the target range of Hb levels in HD patients at 10–12 g/dL in the blood samples collected before the HD session at the beginning of the week [8]. This target range was established based on several studies investigating the association of Hb levels with mortality, CVD, frequency of blood transfusion, and quality of life [9–14].

However, these guidelines are largely based on studies conducted up to the 2000s, and they may not fully align with the current circumstances surrounding dialysis therapy. Since that study era, several changes have occurred in dialysis management in Japan. For example, the improvement in dialysis fluid quality [15], the widespread use of hemodiafiltration (HDF) [16, 17], as well as the dissemination of long-acting ESA, can impact both anemia management and patient prognosis. The rapid increase in aging patients [15] may also potentially influence anemia management. Therefore, uncertainty exists on whether the target Hb levels recommended by these guidelines are applicable to current clinical practice. Furthermore, the optimal Hb level may differ based on the demographic characteristics of patients. Existing studies have indicated that several factors like diabetes, CVD, and older age can modify the association between Hb levels and adverse outcomes in HD patients [11, 18–20], reinforcing the need for further investigation into individualizing the target Hb range.

In this study, we aimed to determine the optimal Hb range applied for current practice and explore the feasibilities of individualizing the target Hb range. We investigated the association between Hb levels and mortality in patients undergoing HD, and explored the potential factors modifying this association using the latest nationwide database.

## Materials and methods

### Study design and population

Data used in this study were obtained from the JSDT Renal Data Registry (JRDR) database from 2019 to 2021. This study involved a nationwide survey of patients undergoing dialysis. Annually, the JSDT collects data from all dialysis units in Japan. The response rate was approximately 95% each year. Details of the JRDR data collection, including the survey items in 2019, have been described elsewhere [15]. Patients undergoing HD thrice a week without combination therapy with peritoneal dialysis (PD) at the end of 2019 were enrolled in this study. Patients who underwent HD for less than 3 h per session, aged < 18 years, with missing data on Hb levels at baseline, and with a dialysis vintage of < 3 months were excluded.

### Exposure of interest and outcomes

The exposure of interest was the Hb level, which was categorized into six groups: < 9.0, 9.0–9.9, 10.0–10.9, 11.0–11.9, 12.0–12.9, and ≥ 13.0 g/dL. The Hb category of 10.0–10.9 g/dL served as the reference in analyses. The primary outcome was all-cause mortality, and the secondary outcomes were cause-specific deaths from cardiovascular, infectious, and malignant diseases. The CVD-related deaths encompassed heart failure, pulmonary edema, ischemic heart disease, arrhythmia, valvular heart disease, cerebral infarction, cerebral hemorrhage, or subarachnoid hemorrhage. Data on deaths were extracted from the records at the end of 2020 and 2021.

### Statistical analysis

Continuous variables were expressed as the mean and standard deviation or median and interquartile range, as appropriate, while categorical variables were expressed as numbers and percentages. Transferrin saturation (TSAT) was calculated by dividing serum iron by total iron-binding capacity. Whole parathyroid hormone (PTH) values were converted to intact PTH values using the following equation: intact PTH = whole PTH × 1.7 [21].

The association between Hb levels and mortality was assessed using Kaplan–Meier (log-rank trend test) and Cox regression analyses. Crude, age- and sex-adjusted, and fully adjusted models were evaluated. The potential confounders at baseline included in the fully adjusted model were as follows: age, sex, body mass index, systolic blood pressure, current smoking status, dialysis vintage, dialysis modality (HD versus HDF), dialysis time per session, fluid removal per body weight, etiology of kidney disease, diabetes, medication for hypertension, history of ischemic heart disease, cerebral hemorrhage, cerebral infarction, and quadruple amputation, single-pool Kt/V, serum levels of creatinine, albumin, total cholesterol, C-reactive protein (CRP), corrected calcium, phosphate, intact PTH and ferritin, TSAT, and use of iron preparations and iron-containing phosphate binders. Variables with skewed distributions, namely, dialysis vintage and levels of CRP, intact PTH and ferritin levels, were log-transformed in the analyses. Dialysis time per session was categorized into three groups: < 4, 4–5, and ≥ 5 h. A fully adjusted restricted cubic spline model with five knots at Hb levels of 9.0, 10.0, 11.0, 12.0, and 13.0 g/dL was used to examine the nonlinear relationship between Hb levels and mortality. Fully adjusted Cox regression analyses were performed to evaluate the cause-specific mortality rate (death from cardiovascular, infectious, and malignant diseases). Subgroup analyses were performed to investigate the consistency of the association between Hb levels and all-cause mortality according to age, sex, dialysis vintage, dialysis modality, dialysis time per session, etiology of kidney disease, diabetes, and history of ischemic heart disease and cerebral infarction. Dialysis vintage was categorized into three groups, closely approximating tertiles. As a sensitivity analysis, stratified Cox regression analysis based on decile of facility-level patient counts was performed to account for differences in practice patterns across facilities. In the stratified Cox model, the difference in facility-level patient counts was accounted for through the variation in baseline hazards across each decile.

Assuming that data were missing at random, multiple imputation by chained equations was employed to impute the missing covariates, leading to the creation of 30 complete datasets. The imputation model included all variables used in the Cox regression analyses, the endpoint indicator, and the Nelson–Aalen cumulative hazard estimate [22]. Results from the 30 datasets were combined using Rubin’s rules [23].

A *P*-value of < 0.05 was considered significant. All statistical analyses were performed using Stata MP 18.0 (Stata Corp., College Station, TX, USA).

## Results

### Baseline characteristics of the study participants

**Fig. 1:**
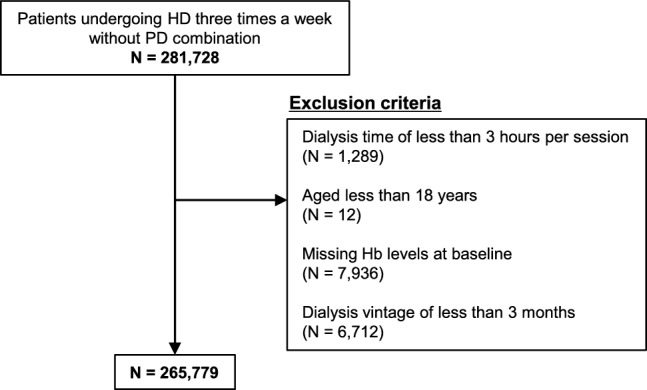
Flowchart diagram showing the study process. *Hb* haemoglobin, *HD* hemodialysis, *PD* peritoneal dialysis

Of the 281,728 patients undergoing HD thrice a week without combination therapy with PD, 265,779 were included in the analyses (Fig. 1). The baseline characteristics according to the Hb category are shown in Table 1. Higher Hb levels were associated with younger age; male sex; higher height; heavier weight; higher frequency of current smoking and HDF; longer dialysis time per session; higher serum levels of urea nitrogen, creatinine, albumin, and phosphate; higher TSAT; and lower serum ferritin levels.

**Table 1:** Baseline characteristics according to the hemoglobin category

	Total	Missing (%)	Hb category (g/dL)					
			< 9	9–9.9	10–10.9	11–11.9	12–12.9	≥ 13
N	265,779		13,423	33,356	82,703	85,008	36,714	14,575
Hb, g/dL	11.0 (1.3)	0	8.2 (0.8)	9.5 (0.3)	10.5 (0.3)	11.4 (0.3)	12.4 (0.3)	13.8 (0.8)
Age, years	69 (12)	0	72 (12)	71 (12)	70 (12)	69 (12)	68 (12)	65 (13)
Male, n (%)	174,679 (65.7)	0	8153 (60.7)	20,706 (62.1)	52,763 (63.8)	56,519 (66.5)	25,584 (69.7)	10,954 (75.2)
Dialysis vintage, months	69 (31–135)	0.05	67 (29–129)	69 (31–132)	68 (32–133)	69 (32–135)	69 (30–137)	78 (34–143)
Etiology of ESKD, n (%)		0						
CGN	69,194 (26.0)		3101 (23.1)	8361 (25.1)	21,512 (26.0)	22,673 (26.7)	9,832 (26.8)	3715 (25.5)
Diabetic nephropathy	104,608 (39.4)		5495 (40.9)	13,454 (40.3)	32,326 (39.1)	32,964 (38.8)	14,425 (39.3)	5944 (40.8)
Nephrosclerosis	31,951 (12.0)		1598 (11.9)	4082 (12.2)	10,218 (12.4)	10,345 (12.2)	4210 (11.5)	1498 (10.3)
PKD	9917 (3.7)		398 (3.0)	1033 (3.1)	2851 (3.4)	3244 (3.8)	1557 (4.2)	834 (5.7)
Others or unknown	50,109 (18.9)		2831 (21.1)	6,426 (19.3)	15,796 (19.1)	15,782 (18.6)	6,690 (18.2)	2584 (17.7)
Height, cm	160.8 (9.6)	6.4	159.2 (9.7)	159.7 (9.7)	160.4 (9.6)	161.1 (9.5)	161.7 (9.5)	163.0 (9.5)
Body weight, kg	57.3 (13.9)	0.8	53.5 (13.1)	55.3 (13.4)	56.9 (13.6)	57.9 (13.9)	58.5 (14.2)	61.0 (15.5)
Body mass index, kg/m^2^	22.0 (4.2)	7.0	21.0 (4.1)	21.5 (4.1)	22.0 (4.1)	22.1 (4.2)	22.2 (4.2)	22.8 (4.6)
Systolic blood pressure, mmHg	152 (24)	1.9	147 (26)	151 (25)	152 (24)	152 (24)	152 (25)	151 (26)
Diastolic blood pressure, mmHg	78 (15)	2.0	74 (15)	76 (15)	78 (14)	79 (14)	80 (15)	82 (15)
Current smoking status, n (%)	23,286 (10.9)	24.4	922 (8.6)	2,575 (9.6)	6960 (10.5)	7592 (11.1)	3591 (12.2)	1646 (14.1)
Dialysis modality, n (%)		0						
HD	144,299 (54.3)		8761 (65.3)	19,499 (58.5)	44,893 (54.3)	44,415 (52.2)	19,185 (52.3)	7546 (51.8)
HDF	121,480 (45.7)		4662 (34.7)	13,857 (41.5)	37,810 (45.7)	40,593 (47.8)	17,529 (47.7)	7029 (48.2)
Dialysis time per session, n (%)		0						
≥ 5 h	25,901 (9.7)		828 (6.2)	2509 (7.5)	7412 (9.0)	8812 (10.4)	4151 (11.3)	2189 (15.0)
4–5 h	202,738 (76.3)		9762 (72.7)	25,101 (75.3)	63,622 (76.9)	65,494 (77.0)	27,980 (76.2)	10,779 (74.0)
< 4 h	37,140 (14.0)		2,833 (21.1)	5,746 (17.2)	11,669 (14.1)	10,702 (12.6)	4583 (12.5)	1607 (11.0)
Fluid removal per body weight, %	4.4 (1.6)	0.8	4.2 (1.8)	4.4 (1.7)	4.4 (1.6)	4.4 (1.5)	4.4 (1.6)	4.3 (1.6)
spKt/V	1.50 (0.32)	4.8	1.43 (0.34)	1.48 (0.33)	1.51 (0.32)	1.51 (0.31)	1.50 (0.31)	1.46 (0.31)
Urea nitrogen, mg/dL	60 (15)	0.3	56 (18)	58 (16)	60 (15)	61 (15)	62 (15)	63 (15)
Creatinine, mg/dL	9.85 (2.82)	0.3	8.29 (2.76)	9.15 (2.72)	9.72 (2.69)	10.10 (2.74)	10.34 (2.87)	10.96 (3.09)
TSAT, %	26.6 (12.9)	21.1	25.4 (17.6)	25.1 (13.7)	26.1 (12.4)	27.0 (12.1)	27.7 (12.7)	28.7 (13.6)
Ferritin, ng/mL	83 (39–163)	7.3	114 (42–254)	94 (39–187)	85 (39–165)	81 (39–154)	76 (39–147)	69 (37–134)
Albumin, g/dL	3.5 (0.4)	0.7	3.2 (0.6)	3.4 (0.5)	3.5 (0.4)	3.6 (0.4)	3.6 (0.4)	3.6 (0.4)
Calcium, mg/dL	8.6 (0.7)	0.3	8.4 (0.8)	8.6 (0.8)	8.6 (0.7)	8.7 (0.7)	8.7 (0.7)	8.7 (0.8)
Phosphate, mg/dL	5.2 (1.5)	0.3	4.7 (1.6)	5.0 (1.5)	5.1 (1.4)	5.3 (1.4)	5.4 (1.5)	5.7 (1.6)
Intact PTH, pg/mL	136 (77–212)	4.7	123 (62–205)	131 (72–209)	135 (77–210)	138 (80–213)	138 (79–217)	142 (78–223)
CRP, mg/dL	0.15 (0.06–0.46)	14.6	0.44 (0.11–1.75)	0.20 (0.07–0.72)	0.15 (0.06–0.44)	0.13 (0.05–0.37)	0.13 (0.06–0.36)	0.16 (0.07–0.41)
Medication for hypertension, n (%)	156,365 (66.6)	13.1	7136 (60.5)	19,530 (66.5)	49,642 (68.0)	51,081 (67.8)	21,406 (65.8)	7570 (58.9)
Diabetes, n (%)	133,014 (54.4)	8.8	7018 (56.6)	17,148 (55.8)	41,138 (54.2)	41,910 (53.7)	18,310 (54.2)	7490 (55.8)
History of ischemic heart disease, n (%)	61,048 (26.9)	17.0	3355 (29.2)	7889 (27.6)	18,705 (26.5)	18,981 (26.1)	8,401 (26.8)	3717 (29.9)
History of cerebral hemorrhage, n (%)	14,661 (6.6)	19.0	918 (8.1)	1963 (7.0)	4449 (6.4)	4532 (6.3)	2004 (6.5)	795 (6.5)
History of cerebral infarction, n (%)	41,520 (18.4)	17.5	2508 (21.9)	5597 (19.6)	12,823 (18.2)	12,744 (17.6)	5645 (18.1)	2203 (17.9)
History of quadruple amputation, n (%)	8520 (3.8)	17.5	629 (5.5)	1238 (4.4)	2418 (3.4)	2478 (3.4)	1139 (3.6)	618 (5.0)

*CGN* chronic glomerulonephritis, *CRP* C-reactive protein, *ESKD* end-stage kidney disease, *Hb* hemoglobin, *HD* hemodialysis, *HDF* hemodiafiltration, *PKD* polycystic kidney disease, *PTH* parathyroid hormone, *spKt/V* single-pool Kt/V, *TSAT* transferrin saturation

### Association of Hb levels with all-cause mortality

**Fig. 2:**
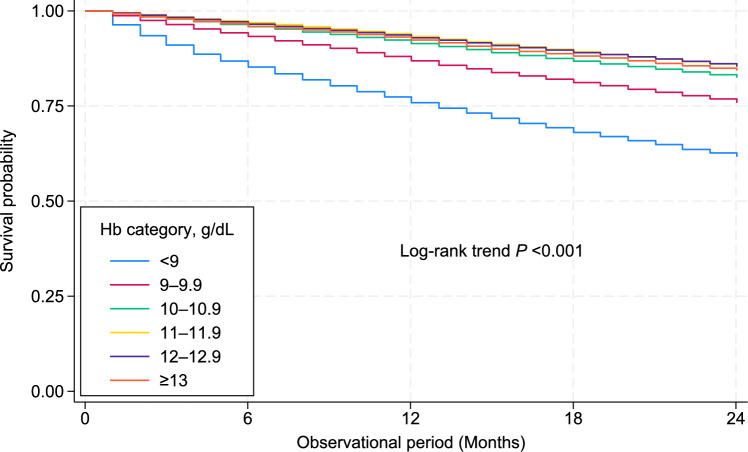
Kaplan–Meier curves for all-cause mortality across all Hb categories. *Hb* hemoglobin

**Fig. 3:**
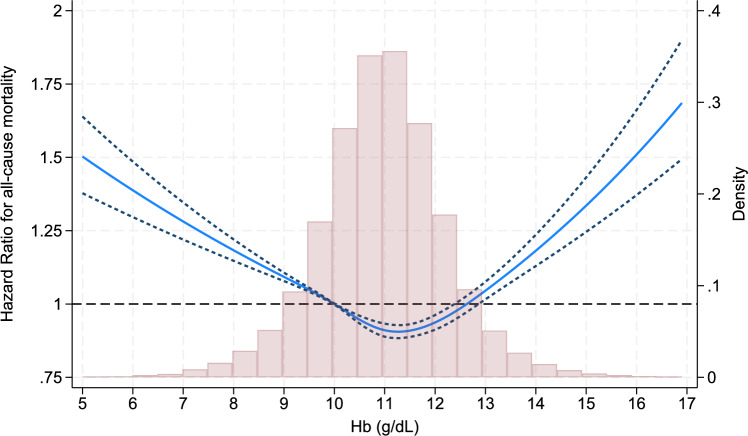
Restricted cubic spline curve depicting the the adjusted hazard ratios and 95% confidence intervals for all-cause mortality, with a reference to an Hb level of 10.0 g/dL, presented alongside a histogram. *Hb* hemoglobin

During a median follow-up period of 24 months, 45,734 patients died, 606 underwent kidney transplantation, and 391 discontinued dialysis. A significant difference was identified in the survival probability across the Hb categories (log-rank trend, *P* < 0.001; Fig. 2). The multivariable Cox regression analysis showed that the Hb categories of < 9.0, 9.0–9.9, and ≥ 13.0 g/dL were significantly associated with higher all-cause mortality compared to the reference category of 10.0–10.9 g/dL, with hazard ratios (HRs) and 95% confidence intervals (CIs) of 1.24 (1.20–1.29), 1.09 (1.06–1.12), and 1.19 (1.14–1.25), respectively (Table 2). The Hb category of 11.0–11.9 g/dL exhibited lower mortality compared to the reference group (HR: 0.95, 95% CI: 0.93–0.98). Restricted cubic spline analysis yielded a consistent result (Fig. 3).

**Table 2:** Hazard ratios and 95% confidence intervals for all-cause mortality and the hemoglobin categories

Model	Hazard ratios (95% confidence intervals)					
	Hb, g/dL					
	< 9	9–9.9	10–10.9	11–11.9	12–12.9	≥ 13
1^a^	2.64 (2.56–2.73)	1.47 (1.43–1.51)	1 (Reference)	0.82 (0.80–0.84)	0.81 (0.79–0.84)	0.87 (0.83–0.91)
2^b^	2.46 (2.38–2.54)	1.41 (1.37–1.45)	1 (Reference)	0.86 (0.84–0.88)	0.90 (0.87–0.93)	1.08 (1.03–1.13)
3^c^	1.24 (1.20–1.29)	1.09 (1.06–1.12)	1 (Reference)	0.95 (0.93–0.98)	1.02 (0.99–1.06)	1.19 (1.14–1.25)

^a^Model 1, crude model

^b^Model 2, adjusted for age and sex

^c^Model 3, Model 2 + adjusted for BMI, systolic blood pressure, current smoking status, dialysis vintage, dialysis modality (HD versus HDF), dialysis time per session, fluid removal per body weight, etiology of kidney disease, diabetes, medication for hypertension, history of ischemic heart disease, history of cerebral hemorrhage, history of cerebral infarction, history of quadruple amputation, single-pool Kt/V, serum levels of creatinine, albumin, total cholesterol, CRP, corrected calcium, phosphate, intact PTH, and ferritin, TSAT, and use of iron preparations and iron-containing phosphate binders

*BMI* body mass index, *CRP* C-reactive protein, *Hb* hemoglobin, *HD* hemodialysis, *HDF* hemodiafiltration, *PTH* parathyroid hormone, *TSAT* transferrin saturation

### Subgroup analysis

Table 3 presents the results of the subgroup analyses. The patients with a dialysis vintage of < 3 years and with a dialysis time of ≥ 5 h did not show an elevated mortality risk in the Hb category of ≥ 13 g/dL. Conversely, those with a dialysis vintage of ≥ 10 years and with a history of cerebral infarction had an increased mortality risk in the higher Hb categories of ≥ 12 g/dL. Regarding the etiology of kidney failure, anemia was not associated with an increased mortality risk in patients with polycystic kidney disease. Other pre-specified relevant factors, including age and dialysis modality, did not substantially modify the relationship between Hb levels and mortality.

**Table 3:** Subgroup analysis of the association between hemoglobin categories and all-cause mortality

	No. of events/total no. of patients	Fully adjusted hazard ratios (95% confidence intervals)					
		Hb, g/dL					
		< 9.0	9–9.9	10–10.9	11–11.9	12–12.9	≥ 13.0
Overall	45,734/265,779	**1.24** **(1.20–1.29)**	**1.09** **(1.06–1.12)**	1 (Reference)	0.95 (0.93–0.98)	1.02 (0.99–1.06)	**1.19** **(1.14–1.25)**
Sex							
Male	30,657/174,679	**1.28** **(1.22–1.33)**	**1.09** **(1.06–1.13)**	1 (Reference)	0.96 (0.93–0.99)	1.02 (0.98–1.06)	**1.15** **(1.09–1.21)**
Female	15,077/91,100	**1.17** **(1.11–1.24)**	**1.08** **(1.03–1.13)**	1 (Reference)	0.95 (0.91–0.99)	1.02 (0.97–1.09)	**1.28** **(1.17–1.40)**
Age, years							
< 65	5,584/83,802	**1.21** **(1.09–1.35)**	**1.13** **(1.03–1.23)**	1 (Reference)	0.88 (0.82–0.95)	1.00 (0.92–1.10)	**1.20** **(1.08–1.34)**
65–74	12,539/86,206	**1.31** **(1.23–1.40)**	**1.13** **(1.07–1.19)**	1 (Reference)	0.94 (0.90–0.99)	1.04 (0.98–1.10)	**1.21** **(1.11–1.32)**
≥ 75	27,611/95,771	**1.18** **(1.13–1.23)**	**1.05** **(1.02–1.09)**	1 (Reference)	0.97 (0.94–1.00)	1.01 (0.97–1.05)	**1.13** **(1.06–1.21)**
Etiology of ESKD							
CGN	9,710/69,194	**1.26** **(1.17–1.36)**	**1.13** **(1.07–1.21)**	1 (Reference)	1.03 (0.98–1.09)	1.05 (0.98–1.13)	**1.22** **(1.10–1.35)**
DN	19,786/104,608	**1.25** **(1.19–1.32)**	**1.09** **(1.04–1.13)**	1 (Reference)	0.94 (0.91–0.98)	1.03 (0.98–1.08)	**1.17** **(1.10–1.26)**
Nephrosclerosis	6,212/31,951	**1.20** **(1.09–1.32)**	**1.14** **(1.05–1.22)**	1 (Reference)	0.99 (0.93–1.06)	1.02 (0.93–1.12)	**1.33** **(1.17–1.52)**
PKD	1,121/9,917	0.97 (0.77–1.22)	0.90 (0.75–1.10)	1 (Reference)	0.91 (0.77–1.07)	1.07 (0.88–1.30)	**1.33** **(1.03–1.71)**
Dialysis modality							
HD	28,353/144,299	**1.22** **(1.17–1.27)**	**1.08** **(1.04–1.12)**	1 (Reference)	0.95 (0.92–0.98)	1.01 (0.97–1.05)	**1.14** **(1.07–1.21)**
HDF	17,381/121,480	**1.29** **(1.22–1.38)**	**1.10** **(1.05–1.15)**	1 (Reference)	0.96 (0.92–1.00)	1.04 (0.99–1.10)	**1.26** **(1.18–1.36)**
Dialysis vintage, years							
< 3	11,492/75,682	**1.26** **(1.18–1.35)**	**1.13** **(1.07–1.20)**	1 (Reference)	0.92 (0.88–0.97)	0.95 (0.89–1.01)	1.09 (0.98–1.20)
3–10	20,635/112,158	**1.25** **(1.19–1.32)**	**1.07** **(1.02–1.11)**	1 (Reference)	0.96 (0.93–1.00)	1.04 (0.99–1.09)	**1.23** **(1.15–1.32)**
≥ 10	13,567/77,805	**1.16** **(1.09–1.24)**	**1.06** **(1.01–1.12)**	1 (Reference)	0.98 (0.94–1.02)	**1.08** **(1.02–1.15)**	**1.23** **(1.13–1.33)**
Dialysis time per session							
≥ 5 h	2,756/25,901	**1.34** **(1.15–1.56)**	1.10 (0.97–1.24)	1 (Reference)	0.85 (0.77–0.94)	0.94 (0.83–1.07)	1.05 (0.89–1.24)
4–5 h	33,061/202,738	**1.25** **(1.20–1.31)**	**1.09** **(1.05–1.13)**	1 (Reference)	0.95 (0.92–0.98)	1.03 (0.99–1.07)	**1.21** **(1.14–1.27)**
< 4 h	9,917/37,140	**1.19** **(1.11–1.27)**	**1.07** **(1.01–1.14)**	1 (Reference)	1.00 (0.95–1.06)	1.05 (0.97–1.12)	**1.21** **(1.08–1.35)**
Diabetes							
Present	25,582/133,014	**1.23** **(1.17–1.28)**	**1.09** **(1.05–1.13)**	1 (Reference)	0.95 (0.92–0.98)	1.03 (0.99–1.08)	**1.18** **(1.11–1.26)**
Absent	16,798/111,328	**1.26** **(1.20–1.34)**	**1.09** **(1.04–1.14)**	1 (Reference)	0.96 (0.92–1.00)	1.01 (0.96–1.06)	**1.20** **(1.11–1.29)**
History of IHD							
Present	14,047/61,048	**1.21** **(1.14–1.28)**	**1.05** **(1.00–1.11)**	1 (Reference)	0.95 (0.91–0.99)	1.03 (0.98–1.09)	**1.18** **(1.10–1.27)**
Absent	25,132/166,053	**1.26** **(1.20–1.32)**	**1.11** **(1.07–1.15)**	1 (Reference)	0.96 (0.93–0.99)	1.01 (0.97–1.06)	**1.19** **(1.12–1.26)**
History of cerebral infarction							
Present	10,985/41,520	**1.27** **(1.19–1.36)**	**1.11** **(1.05–1.17)**	1 (Reference)	1.02 (0.97–1.07)	**1.08** **(1.02–1.16)**	**1.20** **(1.09–1.32)**
Absent	28,151/184,584	**1.23** **(1.18–1.29)**	**1.08** **(1.04–1.11)**	1 (Reference)	0.93 (0.90–0.96)	1.00 (0.96–1.04)	**1.18** **(1.12–1.25)**
CRP, mg/dL							
≥ 0.15	28,467/117,915	**1.25** **(1.20–1.30)**	**1.09** **(1.05–1.12)**	1 (Reference)	0.95 (0.92–0.98)	1.02 (0.98–1.07)	**1.17** **(1.11–1.24)**
< 0.15	11,923/113,908	**1.46** **(1.35–1.58)**	**1.13** **(1.07–1.19)**	1 (Reference)	0.96 (0.91–1.00)	1.02 (0.96–1.08)	**1.23** **(1.13–1.34)**

The bold notations of hazard ratios and 95% confidence intervals indicate that the Hb category is associated with a higher risk of mortality compared with the reference group

*CGN* chronic glomerulonephritis, *DN* diabetic nephropathy, *ESKD* end-stage kidney disease, *Hb* hemoglobin, *HD* hemodialysis, *HDF* hemodiafiltration, *IHD* ischemic heart disease, *PKD* polycystic kidney disease

### Cause-specific death

A total of 14,203, 9714, and 3905 patients died from cardiovascular, infectious, and malignant diseases, respectively. The results of the fully adjusted cause-specific Cox regression analyses are presented in Table 4. The Hb categories of < 9.0, 9.0–9.9, and ≥ 13.0 g/dL were associated with increased CVD mortality compared to the reference category of 10.0–10.9 g/dL. Low Hb categories were associated with an increased risk of death from infectious and malignant diseases, while high Hb categories were not. Subgroup analyses stratified by clinically relevant factors for CVD mortality were also performed (**Supplementary Table 1**). Patients with a dialysis vintage of < 3 years and with a dialysis time of ≥ 5 h showed no increased risk of CVD death in the highest Hb category of ≥ 13 g/dL. However, those with a history of cerebral infarction and with a CRP level of ≥ 0.15 mg/dL had an increased mortality risk in the higher Hb categories of ≥ 12 g/dL.

**Table 4:** Adjusted hazard ratios and 95% confidence intervals for cause-specific death and the hemoglobin categories

Adjusted hazard ratios (95% confidence intervals)					
Hb, g/dL					
< 9	9–9.9	10–10.9	11–11.9	12–12.9	≥ 13
Cardiovascular disease					
1.20 (1.12–1.27)	1.09 (1.04–1.15)	1 (Reference)	0.95 (0.91–0.99)	1.04 (0.99–1.10)	1.32 (1.22–1.43)
Infectious disease					
1.14 (1.06–1.23)	1.01 (0.95–1.07)	1 (Reference)	0.95 (0.90–1.00)	1.00 (0.93–1.07)	1.11 (0.99–1.23)
Malignant disease					
1.64 (1.47–1.82)	1.12 (1.02–1.23)	1 (Reference)	0.87 (0.80–0.94)	0.89 (0.79–1.00)	0.90 (0.75–1.07)

The adjusted variables remained consistent with those utilized in the main analysis: age, sex, body mass index, systolic blood pressure, current smoking status, dialysis vintage, dialysis modality (hemodialysis versus hemodiafiltration), dialysis time per session, fluid removal per body weight, etiology of kidney disease, diabetes, medication for hypertension, history of ischemic heart disease, history of cerebral hemorrhage, history of cerebral infarction, history of quadruple amputation, single-pool Kt/V, serum levels of creatinine, albumin, total cholesterol, C-reactive protein, corrected calcium, phosphate, intact parathyroid hormone, and ferritin, transferrin saturation, and use of iron preparations and iron-containing phosphate binders

*Hb* hemoglobin

### Sensitivity analysis

To account for the difference in facility size, the stratified Cox regression analysis based on deciles of facility-level patient counts was performed. This analysis yielded results consistent with the main analysis (Supplementary Table 2).

## Discussion

In this nationwide study, a clear association was established between Hb levels and mortality in patients undergoing HD. Compared to the Hb category of 10.0–10.9 g/dL, the Hb categories of < 9.0, 9.0–9.9 and ≥ 13.0 g/dL were associated with a higher risk of all-cause mortality. The subgroup analysis further revealed an increased mortality risk associated with the Hb category of 12.0–12.9 g/dL in patients with a dialysis vintage of ≥ 10 years and with a history of cerebral infarction. This finding suggests potential variations in the upper limit depending on the patients’ background. In the JSDT guidelines for renal anemia revised in 2015, the recommended Hb range was 10–12 g/dL [8]. Despite the recent alterations in the background and management of dialysis patients, our results were compatible with the recommendation of the JSDT guideline. This study strengthens the evidence for maintaining an Hb range of 10–12 g/dL in patients undergoing HD.

The observed association between low Hb levels and mortality risk aligns with the report of previous observational studies [9, 10, 24, 25]. Similar to our study, an Hb level below 10–11 g/dL was associated with a higher mortality in these studies. Therefore, establishing a lower limit of the Hb level of 10 g/dL is reasonable. Conversely, an optimal Hb level exceeding 10 g/dL may be considered for minimizing mortality risk. The mortality risk was lowest in the Hb range of 11–12 g/dL, as reported in previous studies [24, 25]. The PARAMOUNT-HD study is a randomized controlled trial that investigated the target Hb levels in Japanese HD patients with hyporesponsiveness to ESA treatment [26]. The proactive treatment group (target Hb level: 11 g/dL) had a lower risk of cardiovascular events compared with the maintenance treatment group (target Hb level: 9–10 g/dL). Although the results of this trial cannot be generalized to all dialysis patients owing to the inclusion criteria, it yielded some insights into the target Hb levels. Further studies are needed to determine the optimal target Hb levels.

This study also provides insights into the upper limit of the Hb level. The normalization of anemia using ESA did not necessarily improve the prognosis in patients with CKD. The Normal Hematocrit Study investigated whether the normalization of hematocrit using epoetin improved the outcomes in patients with congestive heart failure or ischemic heart disease undergoing HD [4]. Compared to the low hematocrit group (hematocrit 30%), the risk of death or nonfatal myocardial infarction increased in the normal hematocrit group (hematocrit 42%), prompting the premature cessation of the study. However, high Hb or hematocrit levels were not necessarily associated with an increased risk of adverse outcomes in observational studies [9–11, 24]. This discrepancy could be attributed to the different settings between the achieved Hb levels in observational studies and the target Hb levels in interventional studies. In observational studies, patients with high achieved Hb levels did not always receive increased doses of ESAs, and some patients had naturally high Hb levels without ESAs. An Hb level of ≥ 12.0 g/dL achieved without ESAs was not associated with an increased risk of mortality in the Dialysis Outcomes and Practice Patterns Study (DOPPS) [27]. However, an Hb level of ≥ 13.0 g/dL was associated with an increased risk of all-cause and cardiovascular death in our study. This finding holds significance in establishing the upper limit of Hb levels. Considering the increased mortality risk observed at an Hb level above 12.0 g/dL in certain subgroups, it seems valid to set the upper limit of Hb levels at 12 g/dL.

Several studies have explored whether the optimal Hb levels depend on the patients’ background. Hanafusa et al. reported that younger patients had poor outcomes at an Hb level of < 10 g/dL, while older patients had poor outcomes at an Hb level of < 9 g/dL, based on the J-DOPPS database [20]. By contrast, our study revealed a consistent relationship between Hb levels and mortality across the age categories. This discrepancy may be owing to differences in the study era or sample sizes between studies. Unlike previous studies, our findings indicated that diabetes and ischemic heart disease did not modify the relationship between Hb levels and mortality. Maekawa et al*.* demonstrated that the presence of CVD or atherosclerosis attenuated the effect of hematocrit levels on mortality risk in HD patients [28]. Furthermore, the relationship between low Hb levels and high mortality risk in dialysis patients diminished among those with diabetes [11, 18]. Currently, it remains unclear whether the lower limit of Hb levels varies depending on the presence of CVD and diabetes, and further studies are needed. Conversely, cerebral infarction influenced the relationship between Hb levels and mortality in our study, with an elevated risk of all-cause and cardiovascular mortality observed at Hb levels above 12 g/dL in those with cerebral infarction. Dialysis sessions were associated with an elevation in whole-blood viscosity, with Hb and hematocrit levels demonstrating a positive correlation with an increase in whole-blood viscosity [29]. Cerebral oxygen delivery was suggested to decrease beyond the hematocrit level of 35% in HD patients undergoing recombinant human erythropoietin therapy [30]. Patients with cerebral infarction may be vulnerable to increased blood viscosity along with elevated Hb levels. We also examined the dialysis background, including dialysis time, vintage, and modality, as stratification factors. Dialysis time per session and modality were associated with anemia and ESA response [31]. In this study, high Hb levels were not associated with an increased mortality risk in patients undergoing dialysis for ≥ 5 h per session. Those with longer dialysis times per session tended to undergo dialysis with lower ultrafiltration rates and have a lower mortality risk [32]. In these patients, the rise in Hb levels may not easily increase blood viscosity. Regarding dialysis vintage, patients with a short dialysis vintage (< 3 years) did not exhibit an elevated risk of mortality at high Hb levels, while those with a long dialysis vintage (≥ 10 years) experienced an increased risk at an Hb level of ≥ 12.0 g/dL. Those undergoing long-term dialysis may be susceptible to increased blood viscosity with elevated Hb levels.

This study demonstrated that Hb levels had different impacts on mortality risk depending on the cause of death, particularly at high Hb levels. The highest category of Hb ≥ 13 g/dL was associated with an increased risk of death due to cardiovascular disease, which aligns with the case of all-cause mortality. This Hb category was also associated with a tendency of a higher risk of death from infectious disease, with HR (95% CI) of 1.11 (0.99–1.23). On the other hand, this association at Hb levels above 13 g/dL was not observed in death from malignant disease. The different impact of high Hb levels on death from malignant disease may be explained by underlying malignant tumor at baseline. Patients with advanced malignant tumor tend to develop anemia and typically do not have high Hb levels. Consequently, it is conceivable that patients with high Hb levels did not have an increased risk of death from malignant disease due to the low probability of having advanced malignant tumor. Further studies incorporating more data on underlying diseases are required regarding the association between Hb levels and cause-specific death.

This study has several limitations. First, we could not establish a causal relationship between Hb levels and outcomes due to its retrospective observational nature. Second, there were unmeasured potential confounding factors. For example, data on ESAs were unavailable in this study. Previous studies indicated that high Hb levels achieved without ESAs did not exacerbate the prognosis in the DOPPS [27]. ESA dose and responsiveness can also influence the results. In addition, data on systemic or hematologic disorders were also unavailable in this study, which could introduce bias. Further studies that account for these factors are needed. Third, as the data from the JRDR survey was collected annually, the variation in Hb levels could not be adequately evaluated in this study. As the variability in Hb levels is associated with the risk of adverse events [33], future studies should incorporate it. Fourth, as we conducted exploratory subgroup analyses across various strata, the results should not be overemphasized. Further studies are needed to confirm the validity of these findings. Finally, the findings of this study may not be applicable to patients taking hypoxia-inducible factor prolyl hydroxylase (HIF-PH) inhibitors. As roxadustat, the first approved HIF-PH inhibitor, was launched in Japan at the end of November 2019, data on HIF-PH inhibitors were unavailable at the study baseline.

In conclusion, the Hb categories of < 9.0, 9.0–9.9, and ≥ 13.0 g/dL were significantly associated with increased risk of mortality compared with the category of 10.0–10.9 g/dL in Japanese patients undergoing HD. The Hb category of 12.0–12.9 g/dL was also associated with an increased risk depending on the patient’s background. We believe that this study strengthens the evidence supporting the optimal Hb range of 10–12 g/dL in patients undergoing HD.

## Supplementary Information

Below is the link to the electronic supplementary material.

Supplementary file1 (DOCX 39 KB)

## Data Availability

Our data are not readily accessible or available to the public due to the constraints imposed by personal information protection laws. The data underlying this article will be shared upon reasonable request from the corresponding authors.
