# Supplementary material for: Association between hemoglobin level and mortality in patients undergoing maintenance hemodialysis: a nationwide dialysis registry in Japan
Source: Clin Exp Nephrol. 2025 Feb 11;29(6):831–42. doi: 10.1007/s10157-025-02632-9 (PMC12125075; doi:10.1007/s10157-025-02632-9)
Supplement: Supplementary file 1 — Supplementary file1 (DOCX 39 KB) [file 10157_2025_2632_MOESM1_ESM.docx]

**Association Between Hemoglobin Level and Mortality in Patients Undergoing Maintenance Hemodialysis: A Nationwide Dialysis Registry in Japan**

**SUPPLEMENTAL MATERIAL**

**List of contents**

**Supplementary Table 1.** Subgroup analysis of the association between hemoglobin categories and cardiovascular mortality.

**Supplementary Table 2.** Hazard ratios and 95% confidence intervals for all-cause mortality and the hemoglobin categories calculated by stratified Cox regression analysis based on decile of facility-level patient counts.

**Supplementary Table 1.** Subgroup analysis of the association between hemoglobin categories and cardiovascular mortality.

|  | No. of events/  total no. of patients | Fully adjusted hazard ratios (95% confidence intervals) | | | | | |
| --- | --- | --- | --- | --- | --- | --- | --- |
|  |  | Hb, g/dL  <9.0 | 9–9.9 | 10–10.9 | 11–11.9 | 12–12.9 | ≥13.0 |
| Overall | 14,203/265,779 | **1.20**  **(1.12–1.27)** | **1.09**  **(1.04–1.15)** | 1 (Ref) | 0.95  (0.91–0.99) | 1.04  (0.99–1.10) | **1.32**  **(1.22–1.43)** |
| Sex |  |  |  |  |  |  |  |
| Male | 9,329/174,679 | **1.20**  **(1.11–1.31)** | **1.09**  **(1.02–1.16)** | 1 (Ref) | 0.96  (0.91–1.01) | 1.03  (0.96–1.11) | **1.29**  **(1.17–1.41)** |
| Female | 4,874/91,100 | **1.18**  **(1.06–1.31)** | **1.10**  **(1.02–1.20)** | 1 (Ref) | 0.93  (0.86–1.00) | 1.05  (0.95–1.16) | **1.34**  **(1.16–1.55)** |
| Age, years |  |  |  |  |  |  |  |
| <65 | 1,961/83,802 | 1.11  (0.92–1.34) | 1.12  (0.97–1.29) | 1 (Ref) | 0.86  (0.76–0.97) | 0.91  (0.79–1.06) | **1.30**  **(1.10–1.54)** |
| 65–74 | 3,995/86,206 | **1.24**  **(1.10–1.40)** | 1.05  (0.95–1.16) | 1 (Ref) | 0.93  (0.85–1.01) | 1.04  (0.94–1.16) | **1.37**  **(1.19–1.57)** |
| ≥75 | 8,247/95,771 | **1.15**  **(1.06–1.25)** | **1.10**  **(1.03–1.17)** | 1 (Ref) | 0.98  (0.93–1.04) | 1.07  (0.99–1.15) | **1.22**  **(1.09–1.37)** |
| Etiology of ESKD |  |  |  |  |  |  |  |
| CGN | 3,797/69,194 | 1.11  (0.97–1.28) | **1.15**  **(1.03–1.29)** | 1 (Ref) | 1.05  (0.95–1.16) | 1.07  (0.95–1.22) | **1.42**  **(1.19–1.68)** |
| DN | 7,488/104,608 | **1.26**  **(1.15–1.38)** | **1.08**  **(1.00–1.17)** | 1 (Ref) | 0.93  (0.87–0.99) | 1.01  (0.93–1.10) | **1.31**  **(1.17–1.46)** |
| Nephrosclerosis | 1,805/31,951 | 1.18  (0.98–1.43) | **1.18**  **(1.03–1.36)** | 1 (Ref) | 1.04  (0.92–1.18) | 1.15  (0.98–1.35) | **1.60**  **(1.28–1.99)** |
| PKD | 328/9,917 | 1.05  (0.67–1.63) | 0.91  (0.63–1.30) | 1 (Ref) | 0.80  (0.59–1.08) | 1.14  (0.81–1.61) | 1.33  (0.85–2.08) |
| Dialysis modality |  |  |  |  |  |  |  |
| HD | 8,700/144,299 | **1.18**  **(1.09–1.27)** | **1.09**  **(1.03–1.17)** | 1 (Ref) | 0.97  (0.91–1.02) | 1.01  (0.94–1.09) | **1.23**  **(1.11–1.37)** |
| HDF | 5,503/121,480 | **1.24**  **(1.11–1.39)** | **1.10**  **(1.01–1.19)** | 1 (Ref) | 0.92  (0.86–0.99) | 1.09  (0.99–1.19) | **1.44**  **(1.28–1.62)** |
| Dialysis vintage, years |  |  |  |  |  |  |  |
| <3 | 3,365/75,682 | **1.25**  **(1.10–1.42)** | 1.10  (0.99–1.22) | 1 (Ref) | 0.97  (0.89–1.06) | 1.00  (0.89–1.12) | 1.12  (0.94–1.34) |
| 3–10 | 6,554/112,158 | **1.20**  **(1.09–1.32)** | **1.13**  **(1.05–1.21)** | 1 (Ref) | 0.94  (0.88–1.00) | 1.06  (0.98–1.16) | **1.33**  **(1.18–1.49)** |
| ≥10 | 4,275/77,805 | 1.10  (0.97–1.23) | 1.02  (0.93–1.12) | 1 (Ref) | 0.96  (0.89–1.04) | 1.07  (0.97–1.19) | **1.46**  **(1.28–1.67)** |
| Dialysis time per session |  |  |  |  |  |  |  |
| ≥5 h | 895/25,901 | **1.46**  **(1.11–1.92)** | 1.20  (0.97–1.49) | 1 (Ref) | 0.90  (0.75–1.07) | 0.89  (0.71–1.12) | 1.23  (0.95–1.61) |
| 4–5 h | 10,301/202,738 | **1.20**  **(1.11–1.30)** | **1.09**  **(1.02–1.15)** | 1 (Ref) | 0.94  (0.89–0.99) | 1.05  (0.99–1.13) | **1.30**  **(1.19–1.43)** |
| <4 h | 3,007/37,140 | 1.12  (0.99–1.28) | 1.09  (0.98–1.21) | 1 (Ref) | 1.00  (0.90–1.10) | 1.06  (0.93–1.21) | **1.38**  **(1.15–1.66)** |
| Diabetes |  |  |  |  |  |  |  |
| Present | 8,319/133,014 | **1.23**  **(1.13–1.33)** | **1.10**  **(1.03–1.17)** | 1 (Ref) | 0.95  (0.89–1.00) | 1.03  (0.95–1.11) | **1.34**  **(1.21–1.47)** |
| Absent | 4,835/111,328 | **1.15**  **(1.03–1.28)** | 1.09  (0.99–1.18) | 1 (Ref) | 0.96  (0.89–1.03) | 1.07  (0.98–1.18) | **1.27**  **(1.11–1.45)** |
| History of IHD |  |  |  |  |  |  |  |
| Present | 4,726/61,048 | **1.14**  **(1.02–1.27)** | 1.05  (0.96–1.14) | 1 (Ref) | 0.93  (0.87–1.00) | 1.01  (0.92–1.11) | **1.33**  **(1.18–1.51)** |
| Absent | 7,375/166,053 | **1.23**  **(1.13–1.34)** | **1.12**  **(1.05–1.20)** | 1 (Ref) | 0.96  (0.91–1.02) | 1.06  (0.98–1.14) | **1.29**  **(1.16–1.43)** |
| History of  cerebral infarction |  |  |  |  |  |  |  |
| Present | 3,489/41,520 | **1.26**  **(1.12–1.43)** | **1.18**  **(1.07–1.30)** | 1 (Ref) | 1.02  (0.93–1.11) | **1.12**  **(1.01–1.25)** | **1.39**  **(1.20–1.62)** |
| Absent | 8,579/184,584 | **1.17**  **(1.08–1.27)** | 1.06  (0.99–1.13) | 1 (Ref) | 0.92  (0.87–0.97) | 1.01  (0.94–1.08) | **1.28**  **(1.16–1.41)** |
| CRP, mg/dL |  |  |  |  |  |  |  |
| ≥0.15 | 8,474/117,915 | **1.21**  **(1.12–1.30)** | **1.11**  **(1.04–1.18)** | 1 (Ref) | 0.97  (0.92–1.03) | **1.08**  **(1.01–1.16)** | **1.38**  **(1.25–1.52)** |
| <0.15 | 3,955/113,908 | **1.34**  **(1.17–1.55)** | 1.09  (0.99–1.20) | 1 (Ref) | 0.91  (0.84–0.98) | 0.98  (0.89–1.08) | **1.20**  **(1.04–1.38)** |

Bold notations of hazard ratios and 95% confidence intervals mean the Hb category is associated with higher risk of mortality compared to the reference group.

Abbreviations: Hb, hemoglobin; ESKD, end-stage kidney disease; CGN, chronic glomerulonephritis; CRP, C-reactive protein; DN, diabetic nephropathy; PKD, polycystic kidney disease; HD, hemodialysis; HDF, hemodiafiltration; IHD, ischemic heart disease.

**Supplementary Table 2.** Hazard ratios and 95% confidence intervals for all-cause mortality and the hemoglobin categories calculated by stratified Cox regression analysis based on decile of facility-level patient counts.

| **Model** | **Hazard ratios (95% confidence intervals)** | | | | | |
| --- | --- | --- | --- | --- | --- | --- |
|  | **Hb, g/dL**  **<9** | **9–9.9** | **10–10.9** | **11–11.9** | **12–12.9** | **≥13** |
| 1 ^a^ | 2.57  (2.48–2.65) | 1.45  (1.41–1.49) | 1 (Ref) | 0.82  (0.80–0.84) | 0.81  (0.79–0.84) | 0.86  (0.83–0.90) |
| 2 ^b^ | 2.41  (2.34–2.49) | 1.40  (1.36–1.43) | 1 (Ref) | 0.86  (0.84–0.88) | 0.90  (0.87–0.93) | 1.07  (1.02–1.12) |
| 3 ^c^ | 1.24  (1.19–1.28) | 1.08  (1.05–1.12) | 1 (Ref) | 0.95  (0.93–0.98) | 1.02  (0.99–1.06) | 1.19  (1.14–1.25) |

^a^Model 1, crude model

^b^Model 2, adjusted for age and sex

^c^Model 3, Model 2 + adjusted for BMI, systolic blood pressure, current smoking status, dialysis vintage, dialysis modality (HD versus HDF), dialysis time per session, fluid removal per body weight, etiology of kidney disease, diabetes, medication for hypertension, history of ischemic heart disease, history of cerebral hemorrhage, history of cerebral infarction, history of quadruple amputation, single-pool Kt/V, serum levels of creatinine, albumin, total cholesterol, CRP, corrected calcium, phosphate, intact PTH, and ferritin, TSAT, and use of iron preparations and iron-containing phosphate binders

Abbreviations: BMI, body mass index; CRP, C-reactive protein; Hb, hemoglobin; HD, hemodialysis; HDF, hemodiafiltration; PTH, parathyroid hormone; TSAT, transferrin saturation
